# Vitamin D Intake, Month the Mammogram Was Taken and Mammographic Density in Norwegian Women Aged 50–69

**DOI:** 10.1371/journal.pone.0123754

**Published:** 2015-05-04

**Authors:** Merete Ellingjord-Dale, Isabel dos-Santos-Silva, Tom Grotmol, Amrit Kaur Sakhi, Solveig Hofvind, Samera Qureshi, Marianne Skov Markussen, Elisabeth Couto, Linda Vos, Giske Ursin

**Affiliations:** 1 University of OsloOslo, Norway; 2 Department of Non-Communicable Disease Epidemiology, London School of Hygiene and Tropical Medicine, London, United Kingdom; 3 Cancer Registry of Norway, Oslo, Norway; 4 University of Southern California, Los Angeles, United States of America; 5 Norwegian Institute of Public Health, Oslo, Norway; 6 Norwegian Knowledge Centre for the Health Services, Health Economic and Drug Unit, Oslo, Norway; 7 Norwegian Centre for Minority Health Research, Oslo, Norway; Geisel School of Medicine at Dartmouth College, UNITED STATES

## Abstract

**Background:**

The role of vitamin D in breast cancer etiology is unclear. There is some, but inconsistent, evidence that vitamin D is associated with both breast cancer risk and mammographic density (MD). We evaluated the associations of MD with month the mammogram was taken, and with vitamin D intake, in a population of women from Norway—a country with limited sunlight exposure for a large part of the year.

**Methods:**

3114 women aged 50–69, who participated in the Norwegian Breast Cancer Screening Program (NBCSP) in 2004 or 2006/07, completed risk factor and food frequency (FFQ) questionnaires. Dietary and total (dietary plus supplements) vitamin D, calcium and energy intakes were estimated by the FFQ. Month when the mammogram was taken was recorded on the mammogram. Percent MD was assessed using a computer assisted method (Madena, University of Southern California) after digitization of the films. Linear regression models were used to investigate percent MD associations with month the mammogram was taken, and vitamin D and calcium intakes, adjusting for age, body mass index (BMI), study year, estrogen and progestin therapy (EPT), education, parity, calcium intakes and energy intakes.

**Results:**

There was no statistical significant association between the month the mammogram was taken and percent MD. Overall, there was no association between percent MD and quartiles of total or dietary vitamin D intakes, or of calcium intake. However, analysis restricted to women aged <55 years revealed a suggestive inverse association between total vitamin D intake and percent MD (p for trend = 0.03).

**Conclusion:**

Overall, we found no strong evidence that month the mammogram was taken was associated with percent MD. We found no inverse association between vitamin D intake and percent MD overall, but observed a suggestive inverse association between dietary vitamin D and MD for women less than 55 years old.

## Introduction

Almost eight decades ago, Peller et al. suggested that sunlight exposure may lower breast cancer risk [1]. In vitro studies have shown that vitamin D has anti-carcinogenic effects, inhibits cell proliferation and angiogenesis in normal and malignant breast cells, and induces cell differentiation and apoptosis [2–4]. There is some, but insufficient, epidemiological evidence regarding the association between vitamin D intake and breast cancer (World Cancer Research Fund 2007). Several case-control studies have reported a significant association between higher vitamin D intake from both diet and supplements and lower breast cancer risk in premenopausal women only [5–8]. However, a recent meta-analysis from the US Preventative Services Task Force found no statistically significant dose-response relationship between 25-hydroxyvitamin D levels and breast cancer risk [9]. Further, the Women’s Health Initiative found no protection of vitamin D and calcium supplements on breast cancer risk, neither during the intervention phase [10] nor 5 years after the invention was stopped [11].

Mammographic density (MD) is an established risk factor for breast cancer. A meta-analysis reported that women with MD of 75% had an increased risk of breast cancer that was 4–5 times higher than in women with low or no density [12]. Although, very dense breasts might mask the tumor until they are larger.

Studies of vitamin D and mammographic density (MD) have also produced inconsistent findings. Some have found high intake of vitamin D to be associated with lower levels of MD [13–17], whereas others have reported no association [18–21]. In a cross-sectional study of Canadian premenopausal women, Brisson et al. reported that changes in blood vitamin D were inversely related to changes in MD, with a lag time of about 4 months [22].

The majority of vitamin D (up to 90%) comes from endogenous production in the skin (vitamin D_3_). Only a small amount of vitamin D comes from food sources, such as dairy products, fatty fish, eggs and butter (vitamin D_2_) [23]. Variation in vitamin D status is due to differences in geographic location, season, sun exposure behavior, sunscreen use, age, skin pigmentation, obesity, and other lifestyle factors [24]. There is a period each year with no or very low cutaneous production of solar ultraviolet B-radiation from 51 degrees north and northwards, which increases in length with latitude [25]. Norway is north of this latitude (58–78º N) and, hence the levels of sunlight exposure during the winter months are low, so dietary intake of vitamin D is necessary to cover the requirements in this period [26,27]. About 35% of the Norwegian female adult population takes cod liver oil (an important source of dietary vitamin D intake) daily in the winter season [28]. The Norwegian diet has traditionally been high in dairy products and fish [29]. Dairy products such as milk and cheese are well-known sources of calcium. Vitamin D and calcium are metabolically interrelated and highly correlated dietary factors that may influence breast cancer risk [30].

In a previous analysis of nutrients and mammographic density from a large subset of the postmenopausal women in these data, we reported no association between vitamin D and mammographic density [31]. However, in that previous study from a study conducted in 2004 we had no women from the furthermost northern county. We therefore decided to expand the study in 2006/2007, to take advantage of the low levels of sunlight exposure in Norway overall, but to also include the women furthest north. We also included premenopausal women from both periods as well as information on month the mammogram was taken. We examined these associations in all Norwegian women aged 50–69 years combined, as well as in strata defined by age (<55 and 55+) and menopausal status. In addition, we examined the association between blood 25-hydroxyvitamin D and MD on a subsample of these women.

## Methods

### Participants

#### 2004 sample

In 2004, a standardized questionnaire on breast cancer risk factors was sent together with the invitation to the Norwegian Breast Cancer Screening Program (NBCSP), the national mammographic screening program of women aged 50–69, to a random sample of 17,050 women living in the counties of Oslo, Akershus and Hordaland. Details about the 2004 study and the characteristics of the study participants have been described previously [31–34]. The questionnaire collected information on menstrual and reproductive history, use of oral contraceptives, menopausal hormonal therapy (HT), family history of breast cancer, and current weight and height. A total of 12,056 (71%) of the invited women attended the screening program and 7,941 (66%) returned a completed questionnaire. A subset (7,174) of the 7,941 women who had completed the risk factor questionnaire was also asked to complete a food frequency questionnaire (FFQ). The FFQ was a 180-item FFQ designed to capture the total energy intake among Norwegian adults the preceding year [31]. It was based on a version of the nationwide NORKOST 1997 survey questionnaire [35]. The estimated nutrient intake was validated against 14-day weighed records (correlation coefficients ranging between 0.42–0.66 [36]. We requested the original mammograms from the various screening centers, prioritizing mammograms from women who had completed the FFQ and who had had a screen film mammography in 2004. About 300 women from Oslo had undergone digital mammography and were not included in the current study as assessments from digital images tend to yield somewhat low percent MD values compared with digitized screen film mammograms (Ursin, unpublished). Women in the 2004 sample were recruited predominantly in the fall of 2004, with a peak in October. We obtained information on risk factors and analogue screening mammograms from 2004 on 2,876 women. Of these, 130 women were excluded for the following reasons: 17 women with previous history of cancer (12 with breast and 5 with ovarian cancers); the breast area could not be determined on mammograms from 3 women; 34 had incomplete data on age; and 73 had incomplete data on BMI (height = 46, weight = 67). Three women were excluded because they used progesterone only, and 19 women had unclassifiable menopausal status (hysterectomy without bilateral oophorectomy). After the exclusions, a total of 2727 (95% = 2727/2876) women with mammograms and dietary data were left for analysis.

#### 2006/07 sample

During 2006/07, we had a separate question in the standardized questionnaire all women receive together with the NBCSP invitation, asking whether they would be willing to participate in an additional study of diet and possibly blood or saliva samples. Information on menstrual and reproductive history, use of oral contraceptive and menopausal hormonal therapy, family history of breast cancer, weight and height were assessed by the NBCSP questionnaire, but questions were similar to those used for the 2004 sample. The FFQ used in the 2006/07 was similar to the one used in 2004, but had been expanded with 4 questions on fruit, 6 on vegetables as well as questions on herbs/nuts/berries to capture variation in antioxidant intake [37]. The FFQ was sent to a random sample of 10,000 women living all over Norway who had agreed to participate in the dietary study. Of these, 6,974 answered the questionnaire and the vast majority (>90%) agreed to provide saliva and a finger prick blood sample. We sent out self-collection kits to 4,597 women and received blood samples from 3,263 women. Mammograms were requested from 5 (in the counties Akershus, Hordaland and Nordland) of the 16 participating screening centers, and we received mammograms for 942 women, out of whom 310 had digital images. We excluded these digital images from the study, leaving us with analogue images for 632 women. We further excluded 7 women with breast cancer, 146 women who had missing data on height and weight and 22 who had missing data on age. Further, 22 women were excluded because of improbable height and weight values (4 with self-reported height<125 cm, and 18 with self-reported weight <30 kg or >170 kg). Five women were excluded because their age was not in the range 50–69 years. Forty-three women were further excluded because of a simple hysterectomy without bilateral oophorectomy (unclassifiable regarding menopausal status). This left us with 387 women to analyze. The final sample of 387 women was the number of women with dietary vitamin D intake with one mammogram.

For the 2006/07 sample, the mammograms were taken throughout the year with a peak in January and March. The final pooled sample size was 3,114 women (N = 2,727 from 2004 and N = 387 from 2006/07).

Compared to the postmenopausal women from 2004 in our previous publication [31], we were able to obtain values for number of pregnancies that had been excluded by Qureshi. Therefore, our number of women differs slightly from this previous paper.

### Blood collection, 25-hydroxyvitamin D analysis

For the 2006/07 sample, we sent a self-collection kit (filter paper, lancets, alcohol pad, alumina bag and a desiccant) and detailed instructions on how to collect finger-tip blood samples by mail. The time range between mammography and blood collection was 2–3 years depending on whether the mammogram was taken in 2006 or 2007. Blood samples were taken from March to June 2009 and 90% of the samples were taken within 8 weeks.

The first two spots on the filter paper were impregnated with a proprietary stabilizing solution (Vitas as, Oslo Norway). The participants were instructed not to eat or drink 10 hours before the fingertip blood sample. Each participant collected blood from a fingertip directly on the filter paper (Whatman 903 paper) and were instructed to have the sample dried for 4–8 hours prior to shipment. The participants were asked to place the filter paper with the dried blood spots (DBS) in an air tight alumina bag together with the desiccant (Whatman, Sanford, USA) and return the DBS cards by regular mail to the study center. The DBS cards were stored at -80°C at the study center.

We sent 403 blood samples for 25-hydroxyvitamin D analyses to a contract laboratory (Vitas AS, Norway). High-performance liquid chromatography (HPLC) Atmospheric Pressure Chemical Ionization (APCI) mass spectrometry (MS) was used to determine 25-hydroxyvitamin D in blood. One hundred and fifty μL of human plasma were diluted with 450 μL 2-propanolcontaining butylated hydroxytoluene (BHT) added as an antioxidant. After thorough mixing (15 min) and centrifugation (10 min, 4000 g at 10°C), an aliquot of 35 μL was injected from the supernatant into the HPLC system. HPLC was performed with a HP 1100 liquid chromatograph (Agilent Technologies, Palo Alta, CA, USA) interfaced by atmospheric pressure chemical ionization to a HP mass spectrometric detector operated in single ion monitoring mode. Vitamin D analogues were separated on a 4.6 mm x 50 mm reversed phase column with 1.8 μM particles. The column temperature was 80°C. A two-point calibration curve was made from analysis of albumin solution enriched with known vitamin D concentration. Recovery was 95%, and the method was linear in the range of 5–400 nM at least. The limit of detection was 1–4 nM. Relative standard deviation (RSD) was 7.6% (47.8 nM) and 6.92 > % (83.0 nM). Coefficient of variation for the analysis was <8%. The lab staff was blinded to the women`s characteristics including their mammographic density readings. Vitas has for several years participated in Vitamin D External Quality Assessment Scheme DEQAS [38] and is evaluated as “Compliant”.

The inclusion criteria for the 25-hydroxyvitamin D analyses were: 8 or more accepted blood spots (out of a possible total of 10), age at mammography > = 50 years, energy intake >2100kJ and <15000 kJ and BMI >15 kg/m^2^ and <50 kg/m^2^. The rationale for these inclusion criteria was to only include women with sufficient blood to conduct other future analysis, and to avoid women in the upper and lower ends of the distributions of these variables.

Of these 403 women we had MD readings for 186 women. Eleven of the blood 25-hydroxyvitamin D samples had missing desiccants. When we excluded these samples, the results remained unchanged (results not shown). Thus, we decided to include the samples with the missing desiccants in the study.

### Vitamin D, calcium and energy intake

Intakes of vitamin D, calcium and energy were estimated on the basis of the data ascertained by the FFQ. The software KBS (version.4.7, 2004) and (version 4.9, 2008) and the Department of Nutrition (University of Oslo) food database were used to calculate the daily intake of energy and nutrients. The food database is based on the official Norwegian food composition table [39]. For vitamin D and calcium we estimated both dietary as well as total (diet plus supplements) intakes.

### Mammographic Density Analysis

We used a high-resolution Kodak Lumisys 85 scanner with automatic feeder to scan the left cranio-caudal analogue mammograms. A computer-assisted method, the Madena software, was used to read the absolute areas of dense and non-dense tissues, as well as percent MD [40]. This method provides a continuous measure of percent MD (calculated by dividing the absolute density by the total breast area and multiplying it by 100) as well as separate estimates of the absolute areas of dense and non-dense tissues. The density assessments for both samples were performed by an experienced reader (G.U.), whereas research assistants trained by G.U. conducted the breast area measurements. The intra-reader correlation coefficient (r^2^) was 0.99 for absolute breast density. The readers were blinded to all subject characteristics.

### Risk factors and menopausal status

BMI was estimated as self-reported weight (in kg) divided by self-reported height (in m^2^). For the 2004 sample, we assessed HT use by asking about ever use and current use of HT with a proposed list of HT preparations. If a woman had used the specified HT for more than three months at the time of completing the questionnaire, she was considered a current user. A woman could have used both estrogen-only (ET) and combined estrogen and progestin therapies (EPT) in her lifetime, but only one of these currently. As for the 2006/07 sample, HT use was assessed by ever, current and past use of HT with a proposed list of HT preparations. We divided the ever HT users into current and past EPT and ET users. Those who were both EPT and ET users were defined as EPT users.

In the questionnaire for the 2004 sample, women were asked whether they had no menstrual period the previous six months and those women were defined as postmenopausal. We also ran a sensitivity analysis excluding women with menopause within the past year (N = 542). Because this yielded essentially unchanged results, we kept this original definition of menopause. Women with hysterectomy without bilateral oophorectomy are unclassifiable with regard to menopausal status, and were therefore excluded. Women in the 2006/07 sample were asked whether they had no menstrual bleeding the previous 12 months, and these women were defined as postmenopausal.

### Statistical analyses

We used multivariate linear regression to examine percent MD associations with dietary and total vitamin D intakes, calcium intake, blood 25-hydroxyvitamin D and month the mammogram was taken (by each month and, in certain analyses, by three broad categories). The majority of the mammograms in 2004 were taken in October to December, and because few women took their mammograms in June to August, September was included in the June to September category.

As the percent MD distribution was right-skewed, we used a square root transformation to normalize it. However, as the regression diagnostics did not improve substantially and, for simplicity, only the results with untransformed MD are presented herein.

The analyses were adjusted for age (continuous), BMI (continuous), HT and EPT use (never, past, current), education (≤10 years, 11–14 years and 15+ years), parity (0, 1, 2 or ≥3), calcium intake (continuous), energy intake (continuous) and study year (2004 or 2006/07). We estimated least squares means (marginal means) on percent MD and all our explanatory variables (age, BMI, HT use, EPT use, educational level, parity, vitamin D, month at mammography, calcium and energy intakes). We ran a test of heterogeneity for whether the association between percent MD and vitamin D intake (total and dietary) differed between the 2004 and 2006/2007 samples, but none of the tests were statistically significant. Therefore, we present the results as one pooled sample.

Several studies have reported a protective effect of vitamin D intake on MD in premenopausal women. We therefore needed to take this into account. Our study included women aged 50–69 at mammography, and we therefore had few women who were premenopausal (N = 452). Because it is not clear why the association might be different in premenopausal women, or exactly when any protective association disappears during menopause, we decided to also stratify by age 55. We therefore investigated the main associations in strata defined by menopause, and in strata defined by age (<55 and 55+). Since this age stratification gave us a larger “young” stratum (N = 1109), and therefore greater statistical power, we present these results in tables, but also mention results for the premenopausal women in the text. We ran a test for heterogeneity to test for effect modification by age <55 and 55+.

We used two sided tests for significance with a p-value <0.05 considered statistically significant, and estimated 95% confidence intervals (CIs). All the analyses were conducted using SAS version 9.2 (SAS Institute, Inc.).

We ran locally weighted polynomial regression models (Proc Loess, SAS) for the 25-hydroxyvitamin D levels with different smoothing parameters (0.2, 0.4, 0.6 and 1). We were not able to find a time trend in vitamin D levels measured in blood, so we present the results without locally weighing.

### Ethics statement

We collected written informed consent from all the participants to use the data provided from the questionnaires, blood samples and mammograms. Data were identified by ID numbers only. The project was approved by the regional ethics committee and the Norwegian Data Inspectorate.

## Results

We have previously described the association between risk factors and percent MD for the 2004 sample [31,34,41,42]. In short, percent MD was inversely associated with age, BMI and parity, but positively associated with educational level, HT use and EPT use (Table 1).

**Table 1:** Percent mammographic density (%MD) by known breast cancer risk factors.

Risk factors	N (3114)	%MD		
		Mean¹	95% CI	
**Age**				
50–54	1015	23.3	22.4	24.2
55–59	793	20.0	18.9	21.0
60–64	626	18.1	16.9	19.2
65–69	393	15.1	13.7	16.6
p for trend		0.0001		
**Body mass index (BMI, kg/m** ^**2**^ **)**				
<25	1574	24.7	23.9	25.4
≥25 - <29	814	16.2	15.2	17.2
≥29	439	10.8	9.4	12.2
p for trend		0.0001		
**Hormone therapy use**				
Never	1429	19.1	18.3	19.8
Ever	1414	21.0	20.2	21.7
p for trend		0.0007		
**Estrogen and progestin therapy use**				
Never	1744	19.2	18.5	19.9
Past	776	20.4	19.4	21.4
Current	307	24.1	22.5	25.7
p for trend		0.0001		
**Number of children**				
0	243	22.9	21.0	24.7
1	333	22.4	20.8	24.0
2	1369	20.2	19.5	21.0
≥3	882	18.2	17.2	19.1
p for trend		0.0001		
**Educational level (years)**				
≤10	976	18.7	17.7	19.6
11–14	873	20.4	19.4	21.3
≥15	978	21.2	20.3	22.2
p for trend		0.0006		

¹Least square means mutually adjusted (for age (continuous), body mass index (continuous), estrogen and progestin therapy (never, past and current), parity (0, 1,2 or ≥3), educational level (≤10 years, 11–14 years and ≥15 years) and study year (0 = 2004 or 1 = 2006/07).

When examining the association in all women, there was a tendency that the lowest MD was seen among those women with mammograms in December and January, and the highest in April (Table 2). However, overall, percent MD was not associated with month the mammogram was taken (p likelihood ratio test = 0.09 =), nor with total vitamin D (p for trend = 0.40), dietary vitamin D (p for trend = 0.96), total calcium intake (p for trend = 0.78) or dietary calcium intake (p for trend = 0.47). Stratifying the analysis by age at mammography (<55 years vs. 55+) revealed an inverse association between total vitamin D intake and percent MD in women <55 years old (p for trend = 0.03). There was, however, little variation in mean percent MD between the three bottom quartiles of the vitamin D intake distribution with the inverse trend reflecting essentially the lower mean percent MD in the top vitamin D quartile (Table 2). Further, the inverse trend between total vitamin D intake and MD was not a convincing dose response trend, because percent MD did not decrease with increasing vitamin D intake (24.8, 24.3, 24.5 and 21.1).

**Table 2:** Mammographic percent density (%MD) by month the mammogram was taken, vitamin D intake (quartiles), and calcium intake (quartiles) in women overall, <55 and > = 55 years at mammography.

	OVERALL				STRATA DEFINED BY AGE AT MAMMOGRAPHY							
	(N = 3114)				<55 (N = 1109)				≥55 (N = 2005)			
Risk factors		%MD				%MD				%MD		
	N	Mean¹	95% CI		N	Mean	95% CI		N	Mean	95% CI	
**Month the mammogram was taken**					** **			** **	** **			
January	55	15.8	11.4	20.3	20	19.0	10.9	27.1	35	13.7	8.4	19.1
February	41	16.8	11.9	21.8	15	16.9	8.0	25.8	16	17.5	11.5	23.4
March	39	16.3	11.2	21.3	12	24.3	14.6	34.1	27	13.1	7.2	18.9
April	94	23.0	20.1	25.9	14	33.1	25.2	41.1	80	19.9	16.9	23.0
May	184	21.8	19.7	23.8	49	25.5	21.2	29.8	135	20.0	17.7	22.4
June	145	20.6	18.2	23.0	62	24.5	20.7	28.3	83	18.8	15.8	21.8
August	16	21.6	14.2	29.0	2	27.2	5.7	48.6	14	19.8	12.1	27.5
September	239	21.7	19.9	23.6	78	25.0	21.6	28.4	161	19.7	17.6	21.9
October	1085	19.9	19.0	20.8	418	23.3	21.8	24.8	667	18.0	16.9	19.1
November	618	20.7	19.5	21.8	241	24.1	22.1	26.0	377	18.5	17.1	19.9
December	18	16.0	8.9	23.0	4	18.1	2.6	33.6	14	13.9	6.2	21.6
P likelihood ratio test^3^		0.09			** **	-0.27				0.21		
**Total vitamin D, μg per day (quartiles)²**												
0 to 5 μg	704	20.3	19.2	21.5	261	24.8	22.9	26.8	443	17.7	16.4	19.1
6 to 8 μg	707	20.1	19.0	21.2	252	24.3	22.4	26.2	455	17.8	16.6	19.1
9 to 12 μg	722	20.3	19.2	21.3	276	24.5	22.6	26.3	446	17.9	16.6	19.2
13 to 49 μg	694	19.5	18.4	20.6	226	21.1	19.0	23.2	468	18.4	17.2	19.7
p for trend		0.40				0.03				0.48		
**Dietary vitamin D, μg per day (quartiles)**												
0 to 3.7 μg	701	20.0	18.8	21.1	257	24.4	22.4	26.5	444	17.4	16.1	18.8
3.71 to 5.7 μg	708	20.2	19.1	21.2	258	23.4	21.5	25.3	450	18.4	17.1	19.7
5.71 to 8.4 μg	716	20.2	19.1	21.3	252	24.2	22.3	26.1	464	17.9	16.7	19.2
8.41 to 38 μg	702	19.9	18.7	21.1	248	23.1	21.0	25.1	454	18.2	16.8	19.6
p for trend		0.96				0.51				0.60		
**Total calcium, mg per day (quartiles)**												
0 to 598 mg	715	20.0	18.8	21.2	259	24.2	22.1	26.4	456	17.8	16.4	19.2
599 to 811 mg	702	20.4	19.3	21.5	248	23.1	21.1	25.0	454	18.6	17.4	19.9
812 to 1062 mg	703	20.1	19.0	21.2	252	23.5	21.6	25.4	451	18.2	17.0	19.5
1063 to 5438 mg	707	19.8	18.6	21.0	256	24.3	22.1	26.5	451	17.3	15.8	18.7
p for trend		0.78				0.93				0.64		
**Dietary calcium, mg per day (quartiles)**												
0 to 575.5 mg	705	20.2	19.0	21.4	246	25.0	22.7	27.2	459	17.6	16.2	19.0
575.6 to 764 mg	703	20.4	19.3	21.4	252	22.3	20.4	24.3	451	19.2	17.9	20.5
765 to 1002 mg	713	20.3	19.2	21.3	250	24.7	22.8	26.6	463	17.8	16.5	19.0
1003 to 5438 mg	706	19.4	18.2	20.7	267	23.2	21.0	25.4	439	17.4	15.8	18.9
p for trend		0.47				0.63				0.57		

¹Least square means mutually adjusted (for age (continuous), body mass index (continuous), estrogen and progestin therapy (never, past and current), parity (0, 1,2 or ≥3), educational level (≤10 years, 11–14 years and ≥15 years) and study year (0 = 2004 or 1 = 2006/07)

Calcium and energy (continuous) (estimated from diet including supplements)

²Total = diet plus supplements

^3^Likelihood ratio test is done on a nested model including variables as described in ^1^ compared to a model including sinusoidal function on month the mammogram was taken.

The suggested inverse results between vitamin D intake and MD in the younger age group, were also observed when analyses were restricted to premenopausal women, with quartile specific estimates of (28.2, 29.1, 28.5 and 24.3 percent MD, p for trend = 0.14, results not shown). In analyses restricted to postmenopausal women only, there was no evidence of percent MD associations with month the mammogram was taken or vitamin D intake (results not shown). There were also no significant associations between percent MD and categories of total and dietary vitamin D intakes defined as low (<10 μg) and high (> = 10 μg) in either group (results not shown). Furthermore, there was no evidence that the magnitude of vitamin D intake (total or dietary) association with percent MD was modified by age (<55 and ≥55 years) (p for heterogeneity = 0.54 for total and 0.76 for dietary vitamin D intake) or menopausal status (p for heterogeneity = 0.86 for total and 0.42 for dietary vitamin D intake, results not shown). We therefore conducted the rest of the analyses for all women combined.

Grouping month the mammogram was taken into three broad categories revealed a positive borderline statistically significant trend between dietary vitamin D intake and percent MD for women whose mammograms were taken in the period of June to September (p for trend = 0.07) (Table 3). Interestingly, an inverse statistically significant association between dietary calcium intake and percent MD (p for trend = 0.04) was also observed among women whose mammograms were taken during the same June-September months. No similar associations were observed for total vitamin D and total calcium intakes in June-September, or in any other period.

**Table 3:** Percent mammographic density (%MD) by quartiles of vitamin D, calcium and energy intakes, stratified by month the mammogram was taken.

	Month the mammogram was taken								
	January-May (N = 497)			June-September (N = 440)			October-December (N = 1860)¹		
**Total vitamin D, μg per day (quartiles)²**	**N**	**%MD³**	**SD**	**N**	**%MD**	**SD**	**N**	**%MD**	**SD**
0 to 5 μg	105	20.1	16.0	105	21.3	16.1	429	20.3	16.1
6 to 8 μg	104	24.7	16.9	82	22.3	19.1	450	19.4	15.8
9 to 12 μg	97	25.9	18.6	106	22.7	14.9	444	19.0	16.3
13 to 49 μg	107	22.7	17.2	107	20.5	13.7	398	18.6	16.2
p for trend		0.28			0.77			0.12	
**Dietary vitamin D, μg per day (quartiles)**									
0 to 3.7 μg	110	21.4	16.1	109	19.6	15.4	414	20.1	15.8
3.71 to 5.7 μg	110	23.9	16.7	80	21.0	17.0	447	19.5	16.0
5.71 to 8.4 μg	110	25.9	17.3	97	22.2	17.5	447	19.2	16.6
8.41 to 38 μg	83	21.6	18.9	114	23.6	15.7	413	18.6	15.9
p for trend		0.50			0.07			0.17	
**Total calcium, mg per day (quartiles)**									
0 to 598 mg	87	21.7	17.9	89	23.4	17.3	472	19.3	15.9
599 to 810 mg	80	22.0	15.0	107	23.2	16.1	428	19.7	16.5
811 to 1062 mg	112	23.9	17.6	103	20.0	16.7	420	19.6	15.8
1063 to 5438 mg	134	24.5	17.8	101	20.2	15.4	401	18.7	16.2
p for trend		0.23			0.10			0.67	
**Dietary calcium, mg per day (quartiles)**									
0 to 575.5 mg	88	23.8	19.0	86	24.7	17.9	465	19.3	15.9
575.6 to 764 mg	91	22.0	16.4	108	22.3	15.7	420	19.9	16.5
765 to 1002 mg	103	22.4	16.0	102	20.4	16.8	432	20.0	16.0
1003 to 5438 mg	131	24.6	17.8	104	19.6	15.2	404	18.1	15.9
p for trend		0.74			0.04			0.47	
**Total energy, KJ per day (quartiles)**									
0 to 6177.5 KJ	91	21.3	17.5	91	18.8	15.8	440	19.4	16.3
6177.6 to 7489 KJ	101	20.8	15.8	103	22.5	17.2	436	19.2	16.3
7489.1 to 8909.5 KJj	101	27.4	17.6	100	21.5	15.9	434	18.3	15.3
8909.6 to 24306 KJ	120	23.4	17.7	106	23.4	16.1	411	20.6	16.5
p for trend		0.08			0.14			0.64	
**Dietary energy, KJ per day (quartiles)**									
0 to 6152 KJ	92	21.3	17.6	89	19.1	15.6	441	19.3	16.2
6152.1 to 7448 KJ	99	20.6	15.8	103	22.4	17.6	434	19.6	16.4
7448.1 to 8858.5 KJ	104	27.3	17.4	102	21.9	16.3	433	18.1	15.3
8858.6 to 24306 KJ	118	23.6	17.8	106	22.8	15.7	413	20.4	16.5
p for trend		0.08			0.202			0.81	

¹The majority of the mammograms in 2004 were taken in October-December. Because there were so few mammograms in June-August we added September to this category

²Total = diet plus supplements

³Least square means adjusted for age (continuous), body mass index (continuous), estrogen and progestin therapy (never, past and current), parity (0, 1,2 or ≥3),

Educational level (≤10 years, 11–14 years and ≥15 years) and study year (0 = 2004 or 1 = 2006/07) energy with supplements (continuous), vitamin D with supplements and calcium (where appropriate).

We stratified women by geographic region of usual residence (67º north versus 60º north). Women from Akershus and Hordaland counties live close to 60º north, and women from the county of Nordland live close to 67º north. There was a suggestive inverse trend between month the mammogram was taken and percent MD in one of the geographic region, but this was not statistically significant (Table 4). The inverse trend persisted after adjustment for vitamin D intake (results not shown).

**Table 4:** Month the mammogram was taken and percent mammographic density (%MD) by geographic region of usual area of residence.

Geographic region¹								
	South (N = 2970)				North (N = 144)			
**Month the mammogram was taken²**	**N**	**%MD³**	**95%Cl**		**N**	**%MD**	**95%Cl**	
January-May	365	20.7	19.1	22.3	48	25.5	21.9	29.2
June-September	378	21.0	19.5	22.4	22	28.7	23.3	34.0
October-December	1687	19.8	19.1	20.5	34	24.5	20.2	28.9
p for trend		0.18				0.81		

¹Usual residence, South, i.e., Akershus and Hordaland counties 60°N and North, i.e., Nordland county 67°N

²n = 268 missing values on month the mammogram was taken(season)

³Least square means adjusted for age (continuous), body mass index (continuous), estrogen and progestin therapy (never, past and current), parity (0, 1,2 or ≥3),

Educational level (≤10 years, 11–14 years and ≥15 years) and study year (0 = 2004 or 1 = 2006/07).

We further investigated the association between total vitamin D intake and percent MD by quartiles of total calcium intake (Table 5). There was no evidence of an association between vitamin D and MD within any quartile of calcium intake. We also examined the association between vitamin D intake and percent MD in non-HT users, but the results were similar to those observed for women overall (results not shown).

**Table 5:** Percent mammographic density (%MD) by total vitamin D intake (quartiles), stratified by total calcium intake (quartiles) (N = 3114).

Total vitamin D intake, μg per day (quartiles)¹					
		0 to 5 μg	6 to 8 μg	9 to 12 μg	13 to 49 μg
Total calcium intake, mg per day (quartiles)	N	% MD Mean²	% MD Mean	%MD Mean	% MD Mean
0 to 598 mg	715	19.9	17.2	19.3	17.9
p for trend					0.34
599 to 811 mg	702	20.2	20.1	20.4	19.3
p for trend					0.70
812 to 1062 mg	703	19.6	21.5	21.0	19.8
p for trend					0.90
1063 to 5438 mg	707	20.1	21.3	21.0	20.7
p for trend					0.96

^1^Total = diet plus supplements

^2^Least square means adjusted for age (continuous), body mass index (continuous), estrogen and progestin therapy (never, past and current), parity (0, 1,2 or ≥3)

Educational level (≥10 years, 11–14 years and 15+ years), study year (0 = 2004 or 1 = 2006/07), calcium with supplements (continuous), and energy with supplements (continuous).

There was a slight positive trend in percent MD with blood levels of 25-hydroxyvitamin D on a subsample of the 2006/07 sample, but with wide 95% CI around the category-specific estimates (Table 6).

**Table 6:** Blood 25-hydroxyvitamin D and percent mammographic density (%MD).

Blood 25-hydroxyvitamin D		%MD		
	N (186)	Mean¹	95%Cl	
<34.9 nmol/L	47	16.4	12.3	20.5
34.91–43.80 nmol/L	46	17.5	13.3	21.6
43.81–52.10 nmol/L	47	17.8	13.7	21.9
52.11–84.61 nmol/L	46	18.6	14.4	22.7
p for trend		0.60		

¹Adjusted by age (continuous), BMI (continuous), calcium and energy intake (continuous) (estimated from diet including supplements).

When we treated the blood levels of 25-hydroxyvitamin D levels as a continuous variable, the results remained the same (beta = 0.03, p = 0.64). We investigated the association between total vitamin D intake and percent MD by quartiles of blood 25-hydroxyvitamin D, but there was no evidence of an association between vitamin D and MD within each quartile of blood 25-hydroxyvitamin D (results not shown).

## Discussion

In our study we found no associations between month the mammogram was taken, vitamin D intake and MD overall, and only a suggested inverse association in women <55 years old.

The daily recommendation of vitamin D intake from the Nordic Nutrition Recommendations 2012 is 10 μg (400 IU) [43]. In the present study, more than 75% of the women who did not take vitamin D supplements, and more than 50% of those who did, had dietary vitamin D intakes lower than the daily recommendations. Given the number of women with low intake, and the range in our sample, we hypothesized that vitamin D intake in our sample could be inversely associated with percent MD.

### Consistency with previous MD studies

Our findings of no overall association of percent MD with vitamin D intake and blood levels are consistent with a number of previous studies. A cohort study of breast cancer families in Minnesota, US, reported no association between dietary vitamin D and percent MD [18]. Neither was there any evidence of an association between dietary vitamin D intake across the life-course and percent MD in a British cohort of women who had been followed regularly from their birth until age 53 [21]. Similarly, no overall effect of vitamin D and calcium supplementation on MD was observed in postmenopausal women enrolled in the Women`s Health Initiative Calcium and vitamin D trial in the US [44]. A case-control study nested within the US Nurses' Health Study found no association between MD and blood 25-hydroxyvitamin D in postmenopausal women [20]. Similarly, no association between 25-hydroxyvitamin D and MD, either percent density or absolute dense area, in a cohort study of breast cancer families in Minnesota, US [19].

Several studies have, however, found an inverse relationship between dietary vitamin D and MD in premenopausal women [13,14,16,17]. In a cross-sectional study on Canadian pre- and postmenopausal women, there was an inverse association between dietary vitamin D and MD among premenopausal, but not postmenopausal, women [15]. Similar results were found in two other studies [13,14]. In our study, when analysis was restricted to women <55 years old, we found a borderline statistically significant association between dietary vitamin D (with supplements) and MD, however the test for heterogeneity between these two age groups was not statistical significant. In a cross-sectional study of Canadian premenopausal women, Brisson and colleagues reported strong seasonal variation in blood levels of 25-hydroxyvitamin D, and modest seasonal variation in MD. Interestingly, the seasonal variation in MD were inversely associated with vitamin D levels, assuming a lag time of about 4 months [22], with lowest MD at the beginning of December, and highest MD early April. Although our findings were not as strong as those of Brisson, we also found the highest MD in April, and the lowest in December/January.

Vitamin D is known to inhibit the mitogenic effects of IGF-I [45]. Epidemiologic and laboratory findings suggest that the IGF pathway may influence the effect of vitamin D and calcium on breast cancer risk and breast density [17]. A cross-sectional study reported an inverse association of dietary vitamin D and calcium intake and mammographic density among Canadian premenopausal women with high insulin growth factor (IGF) levels [17].

When we examined the association between vitamin D intake and MD by month the mammogram was taken, we observed a positive association among those whose mammograms were taken in June through September. This was only observed in analysis of dietary vitamin D. This may therefore have been a chance finding.

### Consistency with previous breast cancer studies

In a systematic review of observational studies and randomized clinical trials assessing associations between vitamin D intake and breast cancer, there was inconclusive evidence for an inverse relationship between vitamin D intake and breast cancer [46]. A meta-analysis of five case–control studies found no overall association between vitamin D intake and breast cancer risk (relative risk = 0.95, 95% confidence interval (CI) = 0.69 to 1.32). However, when they limited the analysis to premenopausal women they found an inverse association with breast cancer risk [47]. Other case-control studies have identified significant associations between vitamin D intake and breast cancer risk in premenopausal women [8,48–50]. Two large cohort studies on Scandinavian women and one report from a large European cohort [51–53], found no evidence of an association between vitamin D intake and risk of breast cancer. One report on French women, found vitamin D intake to be associated with lower breast cancer risk only in those women living in regions with the highest ultraviolet exposure (hazard ratio = 0.68, 95% CI = 0.54–0.85) [54]. In several prospective nested case-control studies investigating the relation between 25-hydroxyvitamin D and breast cancer incidence [55–58], only one reported a significant association between 25-hydroxyvitamin D and breast cancer incidence (odds ratio = 0.73, 95% CI = 0.55–0.96), with the strongest association in women aged <53 years [58]. Finally, the Women’s Health Initiative clinical trial reported no protective effect of the vitamin D and calcium supplement intervention on breast cancer risk [10,11]. Consistent with this, the 2011 Institute of Medicine Committee on dietary intake requirements for calcium and vitamin D in North America concluded that although these vitamins are important for bone health, they have no other health benefits [59].

### Mechanisms; vitamin D and MD

If diet has an effect on mammographic density, this could be through a direct effect of the nutrients on breast tissue [60]. Several in vitro studies have reported that 1.25-dihydroxyvitamin D (the active vitamin D metabolite) may inhibit cellular proliferation and promote differentiation in normal breast tissue and in tumor tissue [61]. Vitamin D and calcium could affect breast cancer risk in part by reducing mammographic density, a strong predictor of breast cancer risk [12,62]. However, as described above, the epidemiologic evidence supporting a protective effect of vitamin D is weak. Our results of no association between vitamin D and MD, but a suggestive protective effect of total vitamin D on MD in women <50 years old, are in line with previous studies.

#### Strengths

In the current study, we investigated the association between vitamin D intake and MD among a population with low sunlight for a large part of the year. Data collection linked to the NBCSP standardized questionnaires, as well as the high intra-reader reliability of the MD readings (intra-reader correlation coefficient r^2^ = 0.99 for absolute breast density) are also strengths of this study.

#### Weaknesses

We had single point measurements of the vitamin D intake, fingerprick blood levels and MD in our cross-sectional study. An issue is how representative these measurements are of women´s vitamin D exposure as well as MD throughout their lives. Vitamin D is known to vary by season and diet. We also used a food frequency questionnaire to assess intake, and it is likely that we have measured this exposure with an error. It is however, unlikely that this error would be differential by MD, or that it would be different by age group. Thus our finding of a suggestive inverse association between total vitamin D intake and percent MD in women aged less than 55 years may be an underestimate. As for MD, a single measure has been found to be relatively stable across ages 50–69, with a decrease of 1–2% per year [63].

Another limitation in the study is the time between the assessment of vitamin D intake and mammographic density measures. For the dietary assessments, the difference in time between assessment of vitamin D intake and mammographic density was 1–2 years, whereas the time between assessment of blood vitamin D intake and mammographic density was 2–3 years. This could partly account for the null results between vitamin D intake and mammographic density.

MD may vary over the menstrual cycle, but we did not have information on menstrual cycle from the premenopausal women in the NBCSP. We have found no reports in the literature that vitamin D varies across the menstrual cycle, therefore this lack of information on day of the menstrual cycle should not represent a problem in our study.

The definition of menopause differed between the 2004 and the 2006/07 samples. This was because of differences in the baseline questionnaires, where the definition in 2004 was based on six months, the definition in 2006/2007 was based on 12 months. However, we ran a sensitivity analysis excluding women with menopause within the past year for the 2004 sample. This yielded mainly unchanged results and we therefore kept the six months definition.

## Conclusion

Overall, we found no evidence of an inverse association between vitamin D and MD, but we observed a suggestive inverse relationship between total vitamin D and MD in women under age 55.

## References

[1] Peller S (1946) Eradication of Breast Cancer, Its Feasibility and Consequences—a Reply. New York State Journal of Medicine 46: 1355–1357. 20988842

[2] Khan QJ, Kimler BF, Fabian CJ (2010) The Relationship Between Vitamin D and Breast Cancer Incidence and Natural History. Current Oncology Reports 12: 136–142. 10.1007/s11912-010-0081-8 20425599

[3] Cui Y, Rohan TE (2006) Vitamin D, calcium, and breast cancer risk: A review. Cancer Epidemiology Biomarkers & Prevention 15: 1427–1437. 10.1158/1055-9965.EPI-06-0075 16896028

[4] Deeb KK, Trump DL, Johnson CS (2007) Vitamin D signalling pathways in cancer: potential for anticancer therapeutics. Nature Reviews Cancer 7: 684–700. 17721433 10.1038/nrc2196

[5] Shin MH, Holmes MD, Hankinson SE, Wu K, Colditz GA, Willett WC (2002) Intake of dairy products, calcium, and vitamin d and risk of breast cancer. J Natl Cancer Inst 94: 1301–1311. 12208895 10.1093/jnci/94.17.1301

[6] Anderson LN, Cotterchio M, Vieth R, Knight JA (2010) Vitamin D and calcium intakes and breast cancer risk in pre- and postmenopausal women. Am J Clin Nutr 91: 1699–1707. 10.3945/ajcn.2009.28869 20392891

[7] Knight JA, Lesosky M, Barnett H, Raboud JM, Vieth R (2007) Vitamin D and reduced risk of breast cancer: a population-based case-control study. Cancer Epidemiol Biomarkers Prev 16: 422–429. 17372236 10.1158/1055-9965.EPI-06-0865

[8] Kawase T, Matsuo K, Suzuki T, Hirose K, Hosono S, Watanabe M, et al (2010) Association between vitamin D and calcium intake and breast cancer risk according to menopausal status and receptor status in Japan. Cancer Science 101: 1234–1240. 10.1111/j.1349-7006.2010.01496.x 20151981 PMC11159182

[9] Chung M, Lee J, Terasawa T, Lau J, Trikalinos TA (2011) Vitamin D With or Without Calcium Supplementation for Prevention of Cancer and Fractures: An Updated Meta-analysis for the US Preventive Services Task Force. Annals of Internal Medicine 155: 827–U883. 10.7326/0003-4819-155-12-201112200-00005 22184690

[10] Chlebowski RT, Johnson KC, Kooperberg C, Pettinger M, Wactawski-Wende J, Rohan T, et al (2008) Calcium plus vitamin D supplementation and the risk of breast cancer. J Natl Cancer Inst 100: 1581–1591. 10.1093/jnci/djn360 19001601 PMC2673920

[11] Cauley JA, Chlebowski RT, Wactawski-Wende J, Robbins JA, Rodabough RJ, Chen Z, et al (2013) Calcium plus vitamin D supplementation and health outcomes five years after active intervention ended: the Women's Health Initiative. J Womens Health (Larchmt) 22: 915–929. 10.1089/jwh.2013.4270 24131320 PMC3882746

[12] McCormack VA, dos Santos Silva I (2006) Breast density and parenchymal patterns as markers of breast cancer risk: a meta-analysis. Cancer epidemiology, biomarkers & prevention: a publication of the American Association for Cancer Research, cosponsored by the American Society of Preventive Oncology 15: 1159–1169. 10.1158/1055-9965.EPI-06-0034 16775176

[13] Thomson CA, Arendell LA, Bruhn RL, Maskarinec G, Lopez AM, Wright NC, et al (2007) Pilot study of dietary influences on mammographic density in pre- and postmenopausal Hispanic and non-Hispanic white women. Menopause 14: 243–250. 17091096 10.1097/01.gme.0000235362.72899.7b

[14] Berube S, Diorio C, Verhoek-Oftedahl W, Brisson J (2004) Vitamin D, calcium, and mammographic breast densities. Cancer Epidemiol Biomarkers Prev 13: 1466–1472. 15342447

[15] Berube S, Diorio C, Masse B, Hebert-Croteau N, Byrne C, Cote G, et al (2005) Vitamin D and calcium intakes from food or supplements and mammographic breast density. Cancer Epidemiol Biomarkers Prev 14: 1653–1659. 16030097 10.1158/1055-9965.EPI-05-0068

[16] Tseng M, Byrne C, Evers KA, Daly MB (2007) Dietary intake and breast density in high-risk women: a cross-sectional study. Breast Cancer Res 9: R72 17949495 10.1186/bcr1781 PMC2242670

[17] Diorio C, Berube S, Byrne C, Masse B, Hebert-Croteau N, Yaffe M, et al (2006) Influence of insulin-like growth factors on the strength of the relation of vitamin D and calcium intakes to mammographic breast density. Cancer Res 66: 588–597. 16397276 10.1158/0008-5472.CAN-05-1959

[18] Vachon CM, Kushi LH, Cerhan JR, Kuni CC, Sellers TA (2000) Association of diet and mammographic breast density in the Minnesota breast cancer family cohort. Cancer Epidemiol Biomarkers Prev 9: 151–160. 10698475

[19] Knight JA, Vachon CM, Vierkant RA, Vieth R, Cerhan JR, Sellers TA (2006) No association between 25-hydroxyvitamin D and mammographic density. Cancer Epidemiology Biomarkers & Prevention 15: 1988–1992. 10.1158/1055-9965.EPI-06-0241 17035410

[20] Green AK, Hankinson SE, Bertone-Johnson ER, Tamimi RM (2010) Mammographic density, plasma vitamin D levels and risk of breast cancer in postmenopausal women. Int J Cancer 127: 667–674. 10.1002/ijc.25075 19960434 PMC2921629

[21] Mishra G, McCormack V, Kuh D, Hardy R, Stephen A, dos Santos Silva I (2008) Dietary calcium and vitamin D intakes in childhood and throughout adulthood and mammographic density in a British birth cohort. Br J Cancer 99: 1539–1543. 10.1038/sj.bjc.6604697 18827811 PMC2579681

[22] Brisson J, Berube S, Diorio C, Sinotte M, Pollak M, Masse B (2007) Synchronized seasonal variations of mammographic breast density and plasma 25-hydroxyvitamin d. Cancer Epidemiol Biomarkers Prev 16: 929–933. 17507618 10.1158/1055-9965.EPI-06-0746

[23] Crew KD (2013) Vitamin d: are we ready to supplement for breast cancer prevention and treatment? ISRN Oncol 2013: 483687 10.1155/2013/483687 23533810 PMC3600307

[24] Giovannucci E (2005) The epidemiology of vitamin D and cancer incidence and mortality: a review (United States). Cancer Causes Control 16: 83–95. 15868450 10.1007/s10552-004-1661-4

[25] Engelsen O, Brustad M, Aksnes L, Lund E (2005) Daily duration of vitamin D synthesis in human skin with relation to latitude, total ozone, altitude, ground cover, aerosols and cloud thickness. Photochem Photobiol 81: 1287–1290. 16354110 10.1562/2004-11-19-RN-375

[26] Macdonald HM, Mavroeidi A, Barr RJ, Black AJ, Fraser WD, Reid DM (2008) Vitamin D status in postmenopausal women living at higher latitudes in the UK in relation to bone health, overweight, sunlight exposure and dietary vitamin D. Bone 42: 996–1003. 10.1016/j.bone.2008.01.011 18329355

[27] Brustad M, Alsaker E, Engelsen O, Aksnes L, Lund E (2004) Vitamin D status of middle-aged women at 65–71 degrees N in relation to dietary intake and exposure to ultraviolet radiation. Public Health Nutrition 7: 327–335. 15003141 10.1079/PHN2003536

[28] Skeie G, Braaten T, Hjartaker A, Brustad M, Lund E (2009) Cod liver oil, other dietary supplements and survival among cancer patients with solid tumours. Int J Cancer 125: 1155–1160. 10.1002/ijc.24422 19444919

[29] Berstad P, Konstantinova SV, Refsum H, Nurk E, Vollset SE, Tell GS, et al (2007) Dietary fat and plasma total homocysteine concentrations in 2 adult age groups: the Hordaland Homocysteine Study. Am J Clin Nutr 85: 1598–1605. 17556699 10.1093/ajcn/85.6.1598

[30] McCullough ML, Rodriguez C, Diver WR, Feigelson HS, Stevens VL, Thun MJ, et al (2005) Dairy, calcium, and vitamin D intake and postmenopausal breast cancer risk in the Cancer Prevention Study II Nutrition Cohort. Cancer Epidemiol Biomarkers Prev 14: 2898–2904. 16365007 10.1158/1055-9965.EPI-05-0611

[31] Qureshi SA, Couto E, Hilsen M, Hofvind S, Wu AH, Ursin G (2011) Mammographic density and intake of selected nutrients and vitamins in Norwegian women. Nutr Cancer 63: 1011–1020. 10.1080/01635581.2011.605983 21916704

[32] Qureshi SA, Couto E, Hofvind S, Wu AH, Ursin G (2011) Alcohol intake and mammographic density in postmenopausal Norwegian women. Breast Cancer Res Treat. 10.1007/s10549-011-1812-8 21993860

[33] Couto E, Qureshi SA, Hofvind S, Hilsen M, Aase H, Skaane P, et al. (2011) Hormone therapy use and mammographic density in postmenopausal Norwegian women. Breast Cancer Res Treat. 10.1007/s10549-011-1810-x 22052325

[34] Ellingjord-Dale M, Lee E, Couto E, Ozhand A, Qureshi SA, Hofvind S, et al (2012) Polymorphisms in hormone metabolism and growth factor genes and mammographic density in Norwegian postmenopausal hormone therapy users and non-users. Breast Cancer Res 14: R135 10.1186/bcr3337 23095343 PMC4053113

[35] Nes M, Frost Andersen L, Solvoll K, Sandstad B, Hustvedt BE, Lovo A, et al (1992) Accuracy of a quantitative food frequency questionnaire applied in elderly Norwegian women. Eur J Clin Nutr 46: 809–821. 1425535

[36] Andersen LF, Solvoll K, Johansson LR, Salminen I, Aro A, Drevon CA (1999) Evaluation of a food frequency questionnaire with weighed records, fatty acids, and alpha-tocopherol in adipose tissue and serum. Am J Epidemiol 150: 75–87. 10400557 10.1093/oxfordjournals.aje.a009921

[37] Qureshi SA, Lund AC, Veierod MB, Carlsen MH, Blomhoff R, Andersen LF, et al (2014) Food items contributing most to variation in antioxidant intake; a cross-sectional study among Norwegian women. BMC Public Health 14: 45 10.1186/1471-2458-14-45 24433390 PMC3902183

[38] DEQAS http://www.deqas.org/.

[39] National Nutrition Council NFCA IfNRU (2001) Den store matvaretabellen (The big food composition table) Norway: Gyldendal Norsk Forlag, ASA.

[40] Ursin G, Ma H, Wu AH, Bernstein L, Salane M, Parisky YR, et al (2003) Mammographic density and breast cancer in three ethnic groups. Cancer epidemiology, biomarkers & prevention: a publication of the American Association for Cancer Research, cosponsored by the American Society of Preventive Oncology 12: 332–338. 12692108

[41] Qureshi SA, Couto E, Hofvind S, Wu AH, Ursin G (2012) Alcohol intake and mammographic density in postmenopausal Norwegian women. Breast Cancer Res Treat 131: 993–1002. 10.1007/s10549-011-1812-8 21993860

[42] Couto E, Qureshi SA, Hofvind S, Hilsen M, Aase H, Skaane P, et al (2012) Hormone therapy use and mammographic density in postmenopausal Norwegian women. Breast Cancer Res Treat 132: 297–305. 10.1007/s10549-011-1810-x 22052325

[43] Recommendations TNN (2012) http://www.norden.org/no/publikasjoner/publikasjoner/2014-002.

[44] Bertone-Johnson ER, McTiernan A, Thomson CA, Wactawski-Wende J, Aragaki AK, Rohan TE, et al (2012) Vitamin D and calcium supplementation and one-year change in mammographic density in the women's health initiative calcium and vitamin D trial. Cancer Epidemiol Biomarkers Prev 21: 462–473. 10.1158/1055-9965.EPI-11-1009 22253296 PMC3297717

[45] Colston KW, Perks CM, Xie SP, Holly JMP (1998) Growth inhibition of both MCF-7 and Hs578T human breast cancer cell lines by vitamin D analogues is associated with increased expression of insulin-like growth factor binding protein-3. Journal of Molecular Endocrinology 20: 157–162. 9513092 10.1677/jme.0.0200157

[46] Chlebowski RT (2011) Vitamin D and breast cancer: interpreting current evidence. Breast Cancer Research 13. 10.1186/bcr2846 PMC3236325 21884640

[47] Chen P, Hu P, Xie D, Qin Y, Wang F, Wang H (2010) Meta-analysis of vitamin D, calcium and the prevention of breast cancer. Breast Cancer Res Treat 121: 469–477. 10.1007/s10549-009-0593-9 19851861

[48] Abbas S, Linseisen J, Chang-Claude J (2007) Dietary vitamin D and calcium intake and premenopausal breast cancer risk in a German case-control study. Nutr Cancer 59: 54–61. 17927502 10.1080/01635580701390223

[49] Lin J, Manson JE, Lee IM, Cook NR, Buring JE, Zhang SM (2007) Intakes of calcium and vitamin D and breast cancer risk in women. Arch Intern Med 167: 1050–1059. 17533208 10.1001/archinte.167.10.1050

[50] Lee MS, Huang YC, Wahlqvist ML, Wu TY, Chou YC, Wu MH, et al (2011) Vitamin D Decreases Risk of Breast Cancer in Premenopausal Women of Normal Weight in Subtropical Taiwan. Journal of Epidemiology 21: 87–94. 21160130 10.2188/jea.JE20100088 PMC3899499

[51] Kuper H, Yang L, Sandin S, Lof M, Adami HO, Weiderpass E (2009) Prospective study of solar exposure, dietary vitamin D intake, and risk of breast cancer among middle-aged women. Cancer Epidemiol Biomarkers Prev 18: 2558–2561. 10.1158/1055-9965.EPI-09-0449 19690185

[52] Edvardsen K, Veierod MB, Brustad M, Braaten T, Engelsen O, Lund E (2011) Vitamin D-effective solar UV radiation, dietary vitamin D and breast cancer risk. Int J Cancer 128: 1425–1433. 10.1002/ijc.25463 20473950

[53] Gonzalez CA, Riboli E (2010) Diet and cancer prevention: Contributions from the European Prospective Investigation into Cancer and Nutrition (EPIC) study. Eur J Cancer 46: 2555–2562. 10.1016/j.ejca.2010.07.025 20843485

[54] Engel P, Fagherazzi G, Mesrine S, Boutron-Ruault MC, Clavel-Chapelon F (2011) Joint effects of dietary vitamin D and sun exposure on breast cancer risk: results from the French E3N cohort. Cancer Epidemiol Biomarkers Prev 20: 187–198. 10.1158/1055-9965.EPI-10-1039 21127286

[55] Bertone-Johnson ER, Chen WY, Holick MF, Hollis BW, Colditz GA, Willett WC, et al (2005) Plasma 25-hydroxyvitamin D and 1,25-dihydroxyvitamin D and risk of breast cancer. Cancer Epidemiol Biomarkers Prev 14: 1991–1997. 16103450 10.1158/1055-9965.EPI-04-0722

[56] McCullough ML, Stevens VL, Patel R, Jacobs EJ, Bain EB, Horst RL, et al (2009) Serum 25-hydroxyvitamin D concentrations and postmenopausal breast cancer risk: a nested case control study in the Cancer Prevention Study-II Nutrition Cohort. Breast Cancer Res 11: R64 10.1186/bcr2356 19715600 PMC2750126

[57] Freedman DM, Chang SC, Falk RT, Purdue MP, Huang WY, McCarty CA, et al (2008) Serum levels of vitamin D metabolites and breast cancer risk in the prostate, lung, colorectal, and ovarian cancer screening trial. Cancer Epidemiol Biomarkers Prev 17: 889–894. 10.1158/1055-9965.EPI-07-2594 18381472 PMC4039037

[58] Engel P, Fagherazzi G, Boutten A, Dupre T, Mesrine S, Boutron-Ruault MC, et al (2010) Serum 25(OH) vitamin D and risk of breast cancer: a nested case-control study from the French E3N cohort. Cancer Epidemiol Biomarkers Prev 19: 2341–2350. 10.1158/1055-9965.EPI-10-0264 20826834

[59] Committee IoM (2011) http://www.iom.edu/~/media/Files/Report%20Files/2010/Dietary-Reference-Intakes-for-Calcium-and-Vitamin-D/Vitamin%20D%20and%20Calcium%202010%20Report%20Brief.pdf.

[60] Brisson J, Verreault R, Morrison AS, Tennina S, Meyer F (1989) Diet, mammographic features of breast tissue, and breast cancer risk. Am J Epidemiol 130: 14–24. 2545096 10.1093/oxfordjournals.aje.a115305

[61] Welsh J (2011) Vitamin D metabolism in mammary gland and breast cancer. Mol Cell Endocrinol 347: 55–60. 10.1016/j.mce.2011.05.020 21669251

[62] Boyd NF, Martin LJ, Yaffe MJ, Minkin S (2006) Mammographic density: a hormonally responsive risk factor for breast cancer. J Br Menopause Soc 12: 186–193. 17178021 10.1258/136218006779160436

[63] Work ME, Reimers LL, Quante AS, Crew KD, Whiffen A, Terry MB (2014) Changes in mammographic density over time in breast cancer cases and women at high risk for breast cancer. Int J Cancer. 10.1002/ijc.28825 PMC4107003 24599445

